# Identification of *Colletotrichum aenigma* as the new causal agent of leaf blight disease on *Aucuba japonica* Thunb., and screenings of effective fungicides for its sustainable management

**DOI:** 10.3389/fmicb.2023.1222844

**Published:** 2023-08-24

**Authors:** Ruidong Fan, Yanjiang Liu, Yalan Bin, Jingyi Huang, Benlin Yi, Xiaoli Tang, Yingxue Li, Yu Cai, Ziyan Yang, Mingxuan Yang, Jiahao Song, Qi Pan, Zengliang Liu, Muhammad Imran Ghani, Xiaojing Hu, Xiaoyulong Chen

**Affiliations:** ^1^College of Agriculture, College of Life Sciences, Guizhou University, Guiyang, China; ^2^International Jointed Institute of Plant Microbial Ecology and Resource Management in Guizhou University, Ministry of Agriculture, China Association of Agricultural Science Societies, Guiyang, China; ^3^Guizhou-Europe Environmental Biotechnology and Agricultural Informatics Oversea Innovation Center in Guizhou University, Guizhou Provincial Science and Technology Department, Guiyang, China; ^4^School of Ecology and Environment, Tibet University, Lhasa, China; ^5^Microbiology Research Institute, Guangxi Agricultural Science Academy, Nanning, China

**Keywords:** *Aucuba japonica*, leaf blight, *Colletotrichum aenigma*, fungicides screening and resistance, agroecological disease

## Abstract

*Aucuba japonica* Thunb is an evergreen woody ornamental plant with significant economic and ecological values. It also produces aucubin, showing a variety of biological activities. It is widely planted in the southwest region of China, including karst landscape areas in Guizhou Province. In January 2022, a serious leaf blight disease was observed on the leaves of *A. japonica* in the outdoor gardens of Guizhou University, Guiyang, Guizhou, China. The causal agent was identified as *Colletotrichum aenigma* through amplification and sequencing of the internal transcribed spacer (ITS) region, translation of the chitin synthase (*CHS*) and actin (*ACT*) genes, and morphological characterizations. Koch’s postulates were confirmed by its pathogenicity on healthy leaves, including re-isolation and identification. To our knowledge, this is the first report of *C. aenigma* causing leaf blight on *A. japonica* worldwide. To identify pathogen characteristics that could be utilized for future disease management, the effects of temperature and light on mycelial growth, conidia production, and conidial germination, and the effects of humidity on conidial germination were studied. Optimal temperatures for mycelial growth of *C. aenigma* BY827 were 25–30°C, while 15°C and 35°C were favorable for conidia production. Concurrently, alternating 10-h light and 14-h dark, proved to be beneficial for mycelial growth and conidial germination. Additionally, conidial germination was enhanced at 90% humidity. *In vitro* screenings of ten chemical pesticides to assess their efficacy in suppressing *C. aenigma* representative strain BY827. Among them, difenoconazole showed the best inhibition rate, with an EC_50_ (concentration for 50% of maximal effect) value of 0.0148 μg/ml. Subsequently, field experiment results showed that difenoconazole had the highest control efficiency on *A. japonica* leaf blight (the decreasing rate of disease incidence and decreasing rate of disease index were 44.60 and 47.75%, respectively). Interestingly, we discovered that *C. aenigma* BY827 may develop resistance to mancozeb, which is not reported yet among *Colletotrichum* spp. strains. In conclusion, our study provided new insights into the causal agent of *A. japonica* leaf blight, and the effective fungicides evaluated provided an important basis and potential resource for the sustainable control of *A. japonica* leaf blight caused by *C. aenigma* in the field.

## 1. Introduction

In the plant world, *Aucuba* (Garryaceae) genus contains 11 evergreen woody species mainly distributed in East and Southeast Asia, including China, Korea, Japan, Myanmar, and Vietnam (Xiang and Boufford, 2005). Some species of this genus, such as *Aucuba japonica* Thunb. and *A. chinensis* Benth., are commonly used as traditional medicines (Kimura et al., 1997; Song et al., 2007). Meanwhile, other species have high economic values as they are usually planted as ornamental plants, for instance, *A. chinensis* Benth., *A. obcordate* (Rehder) Fu ex W.K. Hu and Soong., and *A. japonica* (Robertson, 2010). Consequently, the evergreen shrub *A. japonica*, renowned for its special variegated leaves often referred to as “spotted laurel,” has been widely introduced and cultivated as a garden plant worldwide, including in Europe and the United States (Ali and Kikuzawa, 2005). In addition, the wood of *A. japonica* can be used for handicrafts in the culture and entertainment industry (Huang et al., 2022).

To date, studies on *A. japonica* have mainly focused on geographic structure, systematic, physiology, and pharmacological effects (Ohi et al., 2003; Xue et al., 2009; WCSP, 2019). Although originally distributed in warm temperate and subtropical moist forests of East Asia, *A. japonica* can tolerate heavy shade, air pollution, poor soils, and other stresses like drought (Ali and Kikuzawa, 2005; Zhang et al., 2011). The branching of *A. japonica* is caused by flower bud formation, and previous studies suggested that flower bud abortion by females may reduce its sexual dimorphism in terms of clonal growth (Abe, 2002). Aucubin, extracted from the leaves of *A. japonica*, is widely reported for its significant antioxidant, anti-inflammatory, and neuroprotective properties (Kim et al., 2014; Wang et al., 2015; Yang et al., 2018). However, plant diseases, mainly caused by bacteria, fungi, and viruses, pose major threats to *A. japonica* growth. For instance, in 2019, an outbreak of a disease with southern blight symptoms was first reported on *A. japonica*, and the pathogenic fungus was identified as *Athelia rolfsi*i (Sun et al., 2022). Similarly, from 2018 to 2021, anthracnose disease became a major disease in *A. japonica*, and the pathogenic fungus was confirmed to be *Colletotrichum boninense* (Liu et al., 2022). In addition, the aucuba ringspot virus (AuRV) caused *A. japonica* to show mild mosaic, vein banding, and yellow ringspot symptoms on the leaves, and the vector of AuRV is predicted to be aphids (Uke et al., 2021). Chemical control, using pesticides to suppress pests, is one of the most common and reliable approaches to managing ornamental plant diseases. By way of illustration, ten different insecticides were evaluated for their efficacies against the false oleander scale, *Pseudaulacaspis cockerelli* (Cooley) on *A. japonica* plants (Carson et al., 2021). However, little is known about active fungicide resources against particular fungal diseases on *A. japonica*. Commonly used fungicides for crops and horticulture include tebuconazole, mancozeb, difenoconazole, pentachloronitrobenzene, and others, which have achieved relatively good inhibitory effects (Homdork et al., 2000; Fan et al., 2016; Wang et al., 2021). Nevertheless, it has been proven that misuse or overuse of pesticides has caused many problems for the environment and the quality and safety of agricultural products (Smith et al., 2013; Yesica et al., 2020). Thus, it is important to screen chemical pesticides with high efficiency, low toxicity, and low residue to control the disease.

Among the *Colletotrichum gloeosporioides* species complex, *C. aenigma* is an agronomically important phytopathogenic fungal species causing anthracnose diseases in a variety of hosts, including cereals, legumes, vegetables, perennial crops, and tree fruits (Than et al., 2008; Damm et al., 2012; Guarnaccia et al., 2017). In general, infections caused by *Colletotrichum* spp. start with spore attachment to the host plant surface, followed by spore germination and the development of an appressorium that penetrates the cuticle of the plant (Moral et al., 2021). The majority of *Colletotrichum* spp. colonies exhibited similar texture and density characteristics, characterized by abundant aerial mycelium with regular margins. These colonies display a wide range of color variations, ranging from white and whitish to dark gray and pinkish-orange (Zheng et al., 2021). The occurrences of plant disease were influenced by environmental factors and management strategies, especially high temperature and humidity, which can lead to enhanced pathogenesis of phytopathogens (Ram et al., 1999; Zhang et al., 2012; Romero et al., 2022). Moreover, Estrada et al. (2000) reported that temperature and humidity variations could affect the pathogenicity, conidial germination, and appressoria formation of *Colletotrichum* spp., which can be used to control anthracnose in the field condition. For this reason, exploring the interactions between different factors such as humidity, temperature, photoperiod, and growth duration is critical for our understanding of the pathogenic mechanism and effective control of *C. aenigma*. The first report of *C. aenigma* causing anthracnose diseases on *Pyrus pyrifolia* was in Japan (Weir et al., 2012). Subsequently, *C. aenigma* was reported as a phytopathogen on a variety of hosts, such as *Pyrus bretschneideri*, *Malus pumila*, and *Vitis vinifera* (Yan et al., 2015; Fu et al., 2019; Lee et al., 2021), resulting in significant yield and quality losses in these plants. To our knowledge, *C. aenigma* has not yet been reported as a pathogen for *A. japonica*.

In general, *C. aenigma* poses a major threat to plants, causing dramatic losses in agriculture, forestry, and fruit production worldwide. The primary means of prevention and control of *Colletotrichum* spp. has been through the use of chemical fungicides in the last decades. However, the excessive use of these chemical fungicides may cause a series of problems over time, such as food safety issues due to pesticide residues (Abrol and Singh, 2003), off-target and side effects on the environment, human health, and other living organisms (Wilson and Tisdell, 2001; Williams et al., 2004; Schmitt-Jansen and Altenburger, 2005). Hence, it is significant to screen effective chemical fungicides to manage newly emerging diseases like leaf blight of *A. japonica* caused by *C. aenigma*. Therefore, the objectives of the present study were to: (1) isolate and identify the potential pathogens that caused serious anthracnose disease on *A. japonica* in Guiyang, Guizhou, China; (2) confirm the pathogenesis of the representative isolates; (3) characterize the biological characteristics of the pathogen referring to local karst landscape environmental factors; and (4) screening effective and sustainable fungicides for the management of the disease.

## 2. Materials and methods

### 2.1. Sample collection and fungal isolation

In January 2022, a serious leaf blight disease was observed on the leaves of *A. japonica* in the outdoor gardens of Guizhou University, Huaxi District, Guiyang City, Guizhou Province, China (26°44′58”N, 106°65′91″E). On the leaf surface of *A. japonica*, a burn-like black blight with wilting signs was frequently observed, leading to rapid and extensive foliage death. The disease affected an area of approximately 0.52 hectares, with disease incidence ranging from 60 to 70%, resulting in a mortality rate of 20 to 30% in plants. For fungal isolation, every two symptomatic leaves were collected from ten different plants using disposable gloves. The diseased leaves were first soaked in 75% v/v ethanol for 1 min, then in a 4% w/v sodium hypochlorite (NaClO) solution for 3 min, and finally washed thrice with sterile distilled water (dH_2_O). Subsequently, the leaves were placed on potato dextrose agar (PDA) medium and incubated at 25 ± 2°C for 7 days. The fungal colonies were purified by transferring single spores, pure cultures were obtained. Representative isolates were selected for further identification.

### 2.2. Morphological and molecular identification

The morphological characteristics of five representative isolates were examined under the microscope. Fungal conidia were observed and photographed using a Zeiss microscopic system (Carl Zeiss-Axioscope 5, Germany). The length and width of 60 randomly selected conidia were measured and recorded. For molecular identification and phylogeny analysis, the genomic DNA of the representative isolates was extracted from mycelia of seven-day-old cultures according to the manufacturer’s instructions (Biomiga Fungal DNA Extraction Kit, CA, United States). Subsequently, polymerase chain reaction (PCR) amplification of internal transcribed spacer (rDNA-ITS), a partial sequence of the chitin synthase (*CHS*) gene and actin (*ACT*) gene were performed using the following primers: ITS1 (5’-TCCGTAGGTGAACCTGCGG-3′) and ITS2 (5′-GCTGCGTTCTTCATCGATGC-3′; White et al., 1990), *CHS*-79F (5′-TGGGGCAAGGATGCTTGGAAGAAG-3′) and *CHS*-345R (5′-TGGAAGAACCATCTGTGAGAGTTG-3′; Carbone and Kohn, 1999), and *ACT*-512F (5′-ATGTGCAAGGCCGGTTTCGC-3′) and *ACT*-783R (5′-TACGAGTCCTTCTGGCCCAT-3′; Carbone and Kohn, 1999), respectively. The PCR amplifications were carried out in a Bio-Rad S1000 Thermal Cycler in a 25 μl reaction mixture containing 1 μl of DNA sample, 1 μl of each primer, 12.5 μl 2 × SanTaq PCR Mix (Sangon Biotech, Shanghai, China), and 9.5 μl double distilled water (ddH_2_O). The PCR conditions were as follows: initial denaturation at 94°C for 3 min, followed by 35 cycles of denaturation at 95°C for 30 s, annealing for 30 s at the corresponding temperatures (53°C for ITS, 58°C for *CHS* gene, and 61°C for *ACT* gene), extension at 72°C for 45 s, then a final extension for 10 min.

The DNA sequences obtained in this study were queried against other DNA sequences in the GenBank database in the National Center for Biotechnology Information (NCBI).^1^ Sequences of the sequenced DNA regions of strains have been deposited with GenBank. The phylogenetic tree was constructed by the neighbor-joining algorithms based on concatenated ITS region, *CHS* gene, and *ACT* gene sequences using MEGA 6.05 software (Tamura et al., 2013).

### 2.3. Pathogenicity test

To confirm Koch’s postulates, the pathogenicity of the representative strain *C. aenigma* BY827 was tested on healthy leaves of *A. japonica*. Firstly, inoculation was performed in a laminar flow workstation (Haier, Qingdao, China) using sterilized needles to perform pathogen inoculation on healthy *A. japonica* detached leaves (*n* = 9; Bajwa et al., 2010). Then, strain *C. aenigma* BY827 plugs (d = 5 mm) were inoculated onto the leaves, obtained from active growing *C. aenigma* BY827 colonies that were 7 days old. As a control, nine leaves were mock-inoculated with sterile non-inoculated PDA medium plugs. After inoculation, all leaves were incubated at 25 ± 2°C, with a 16 h: 8 h photoperiod, and 70 ± 2% relative humidity (RH). In parallel, the pathogenicity of the representative strain *C. aenigma* BY827 was verified on each of six leaves of three healthy 5-year-old *A. japonica* plants grown in the campus green space of Guizhou University, Guiyang, Guizhou, China. Each leaf was spray inoculated with 500 μL conidial suspension (1 × 10^5^ conidia/mL) of *C. aenigma* BY827. Another three plants sprayed with equal amounts of sterile distilled water served as controls. The pure cultures of the pathogen were reisolated from diseased *A. japonica* leaves and confirmed through the molecular analysis mentioned above. The experiment was repeated three times.

### 2.4. The effect of temperature on mycelial growth, conidia production, and conidial germination rate

To study the mycelial growth and conidia production of *C. aenigma* BY827 at different temperatures, a 5 mm diameter mycelial plug was taken from the edge of a seven-day-old colony and placed in the center of a petri dish with PDA medium and then incubated at different temperatures. The petri dishes were then incubated at temperatures, ranging from 15°C to 35°C at 5°C intervals. Mycelial growth was recorded on the 5th day after inoculation. Three repetitions were conducted for each treatment, and the fungal colony’s diameters were measured by the criss-cross-method (Hu et al., 2022). Ten agar-mycelium plugs (5 mm diameter) were taken from the edge of the active growing colonies simultaneously and transferred into a centrifuge tube containing sterile water. The solution was vigorously vortexed and then filtered through a double-layer gauze. Afterwards, the solution was diluted, and a hemocytometer was used to count the number of conidia per square centimeter of the colony, which was calculated using the formula below. For the germination rate analysis, conidia from seven-day-old colonies were washed with sterile distilled water, and the conidia concentration was adjusted to 1 × 10^5^ conidia/mL using a hemocytometer. Subsequently, 0.2 mL of conidial suspension was added to 0.2 mL potato dextrose broth (PDB). After 12 h, the germination rate was calculated by microscopy.


(1) $$\mathrm{Conidia production}\;\left(\mathrm{conidia}/{\mathrm{cm}}^{2}\right)=X\times N\times 5\times 10^{4}/n\pi r^{2}\;$$

X: dilution fold, N: the number of conidia in five squares of the hemacytometer, n: the number of perforated agar-mycelium plugs, r: the inner diameter of the perforator (Deng et al., 2012; Cao et al., 2022).

### 2.5. The effect of photoperiod on mycelial growth, conidia production, and conidial germination rate

To assay the effects of photoperiod on *C. aenigma* BY827, inoculated growth medium (PDA for mycelial growth and conidia production; PDB for conidial germination rate) was placed in incubators at 25 ± 2°C with the following settings: (1) 6 h light and 18 h dark; (2) 10 h light and 14 h dark; (3) 14 h light and 10 h dark; (4) 18 h light and 6 h dark. Each treatment was performed in triplicates.

### 2.6. The effect of humidity on conidial germination rate

The experiment aimed to determine the effect of humidity on the conidial germination rate of *C. aenigma* BY827. A conidial suspension (1 × 10^5^ conidia/mL) was smeared onto glass slides, dried with sterile air, and placed in a closed petri dish. The relative humidity was set to 60, 70, 80, and 90%, respectively. The plates were incubated for 24 h at 25 ± 2°C in the dark, and the conidial germination rate was determined by microscopic observation after 12 h. Each treatment was repeated three times.

### 2.7. *In vitro* antifungal activity of fungicides on mycelial growth

Antifungal activities of eight chemical fungicides and two biopesticides against a representative strain of *C. aenigma* BY827 were screened to explore sustainable control resources for managing the pathogen. The mycelial growth rate method was used (Xin et al., 2020). The employed fungicides were formulations of tebuconazole (43% suspension concentrate; SC), mancozeb (43% SC), difenoconazole (10% water-dispersible granule; WG), pentachloronitrobenzene (40% dust powder; DP), myclobutanil (12.5% emulsifiable concentrates; EC), dimethachlone (40% wettable powder; WP), hymexazol (70% WP), thiram (50% WP), carvacrol (5% aqueous solutions; AS), and kasugamycin (2% AS). They were purchased from a local distributor and diluted to various concentrations for further experiments (Table 1). Different fungicides were dissolved in organic solvents or water (myclobutanil was dissolved in acetone, and all other fungicides were dissolved in water). For those fungicides dissolved in acetone, a comparable final concentration of the indicated solvents was added into the PDA medium, which served as control plates (CK). Different concentrations of fungicides were mixed with PDA medium, and 10 mL was dispensed into sterile 9 cm diameter petri dishes. Pathogenic mycelial plugs of representative strain *C. aenigma* BY827 (5 mm in diameter) were inoculated into the center of PDA plates containing varying concentrations of gradient agents, and each treatment was repeated three times. The plates were then cultured at 25 ± 2°C under dark conditions. After 7 days, the colony diameter of each treatment was measured. The following equation was used to determine the inhibition efficiency of the fungicides, and EC_50_ (concentration for 50% of maximal effect) values were calculated.


(2) $$R\;\left(\%\right)=\frac{\left(C-T\right)}{\left(C-F\right)}\times 100\%$$

R: relative inhibition rates, C: diameter of the fungus in control, T: diameter of the fungus in the treatment, F: diameter of fungus plugs (Luo et al., 2021).

**Table 1:** Concentrations of substances used for fungicide sensitivity assays and their China pesticide registration numbers (http://www.chinapesticide.org.cn).

Fungicide name	Registration number	Active ingredient	Manufacturer	Substance concentration (μg/mL)
Tebuconazole 43% SC	PD20050216	Tebuconazole	Bayer AG, Germany	0.43, 0.215, 0.1075, 0.05375, 0.026875
Mancozeb 43% SC	PD20081132	Mancozeb	Dow AgroSciences, United States	6.88, 3.44, 1.72, 0.86, 0.43
Difenoconazole 10% WG	PD20152176	Difenoconazole	Syngenta Nantong Crop Protection Co., Ltd. China	0.1, 0.05, 0.025, 0.0125, 0.00625
Pentachloronitrobenzene 40% DP	PD20060171	Pentachloronitrobenzene	Sichuan Runer Technology Co., Ltd. China	0.8, 0.4, 0.2, 0.1, 0.05
Myclobutanil 12.5% EC	PD20086370	Myclobutanil	Shenzhen Noposion Agrochemicals Co., Ltd. China	1, 0.5, 0.25, 0.125, 0.0625
Dimethachlone 40% WP	PD20150266	Dimethachlone	Jiangxi Heyi Chemical Co., Ltd. China	12.8, 3.2, 0.8, 0.2, 0.05
Hymexazol 70% WP	PD20100877	Hymexazol	Tianjin Luheng Chemical Co., Ltd. China	2.8, 1.4, 0.7, 0.35, 0.175
Thiram 50% WP	PD20093058	Thiram	Shandong Bainong Sida Biotechnology Co., Ltd. China	8, 4, 2, 1, 0.5
Carvacrol 5% AS	PD20200138	Carvacrol	Shanxi De Wei Materia Medica Biologic Biotechnology Co., Ltd. China	1.6, 0.8, 0.4, 0.2, 0.1
Kasugamycin 2% AS	PD54-87	Kasugamycin	Hokko Chemical Industry Co., Ltd. Japan	1.28, 0.64, 0.32, 0.16, 0.08

SC, suspension concentrate; WG, water-dispersible granule; DP, dust powder; EC, emulsifiable concentrates; WP, wettable powder; AS, aqueous solutions.

### 2.8. Field experiments

Four pesticides, namely difenoconazole, tebuconazole, pentachloronitrobenzene, and mancozeb, were selected to determine their effectiveness in controlling the leaf blight disease caused by *C. aenigma* in *A. japonica*. The experiments were carried out at the campus green space of Guizhou University, Guiyang, Guizhou, China, which was naturally infested with *A. japonica* leaf blight. The pesticides were formulated according to the recommended concentration for field use. To conduct the experiment tebuconazole (at a concentration of 2.87 μg/mL in sterile water containing 43% active pesticide ingredients), mancozeb (at a concentration of 10.75 μg/mL in sterile water containing 43% active pesticide ingredients), difenoconazole (at a concentration of 1.67 μg/mL in sterile water with 10% active pesticide ingredients), and pentachloronitrobenzene (at a concentration of 266.67 μg/mL in sterile water with 40% active pesticide ingredients) were used as the pesticide treatments, while sterile water was used as the control treatment. Each treatment was repeated three times. A pump sprayer was used to evenly spray the 5-year-old plants of *A. japonica*. The volume of sprayed liquid was 5 mL per plant. Leaf blight was investigated before and 14 days after spraying. A total of 100 plants were randomly selected in each treatment (6 m^2^). According to Fang (1998), the disease grading was based on the percentage of disease spots on leaves in the whole leaf area: Grade I, healthy, representative value was 0; Grade II, diseased area ≤ 25%, representative value was 1; Grade III, diseased area > 25 and ≤ 50%, representative value was 2; Grade IV, diseased area > 50 and ≤ 75%, representative value was 3; and Grade V, diseased area > 75%, representative value was 4. Meanwhile, the disease incidence, disease index, and control efficiency were calculated using the following formula (Henderson and Tilton, 1955; Sun and Song, 2002):


(3) $$\begin{array}{l} \mathrm{Disease incidence}\;\left(\%\right)=\\ \frac{\left(\sum \mathrm{number of diseased leaves of each grade}\right)}{\mathrm{total number of leaves investigated}}\times 100\% \end{array}$$

(4) $$\begin{array}{l} \mathrm{Control efficiency}\;\left(\mathrm{Decreasing rate of disease incidence}\right)\\ \;\;\;\;\;\;\;\;\;\;\;\;\;\;\;\;\;\;\;\;\;\;\;\;\;\;\;\;\;\;\;\;\;\;\;\;\;\;\;\;\;\;\;\;\;\left(\%\right)=\frac{\left({\mathrm{pt}}_{0}\times {\mathrm{pt}}_{1}\right)}{{\mathrm{pt}}_{0}}\times 100\% \end{array}$$

pt_0_: disease incidence in pesticide treatment plot before spraying, pt_1_: disease incidence in pesticide treatment plot after spraying.


(5) $$\begin{array}{l} \mathrm{Disease index}=\\ \frac{\left[\sum \left(\begin{array}{l} \mathrm{number of diseased leaves of each grade}\times \\ \mathrm{disease grade} \end{array}\right)\right]}{\left(\begin{array}{l} \mathrm{total number of leaves investigated}\times \\ \mathrm{the highest disease index} \end{array}\right)}\times 100 \end{array}$$

(6) $$\begin{array}{l} \mathrm{Control efficiency}\;\left(\mathrm{Decreasing rate of disease index}\right)\\ \left(\%\right)=\left[1-\frac{\left({\mathrm{CK}}_{0}\times {\mathrm{PT}}_{1}\right)}{\left({\mathrm{CK}}_{1}\times {\mathrm{PT}}_{0}\right)}\right]\times 100\% \end{array}$$

CK_0_: disease index in control plot before spraying, CK_1_: disease index in control plot after spraying, PT_0_: disease index in pesticide treatment plot before spraying, PT_1_: disease index in pesticide treatment plot after spraying.

### 2.9. Statistical analysis

All percentage data were subjected to arcsine transformation and expressed as the mean ± SE (standard error of the mean). The statistical significance of the results was calculated using a one-way analysis of variance followed by Duncan’s new complex polar difference method at a significant level of *p <* 0.05 using IBM SPSS Statistics software version 25.0. The EC_50_ of different fungicides was calculated with a toxicity regression equation using the Data Processing System (DPS) v7.05 software (Pasche et al., 2004). Figures were created using GraphPad Prism (v8.0.2) software.

## 3. Results

### 3.1. Morphological characterization

**Figure 1:**
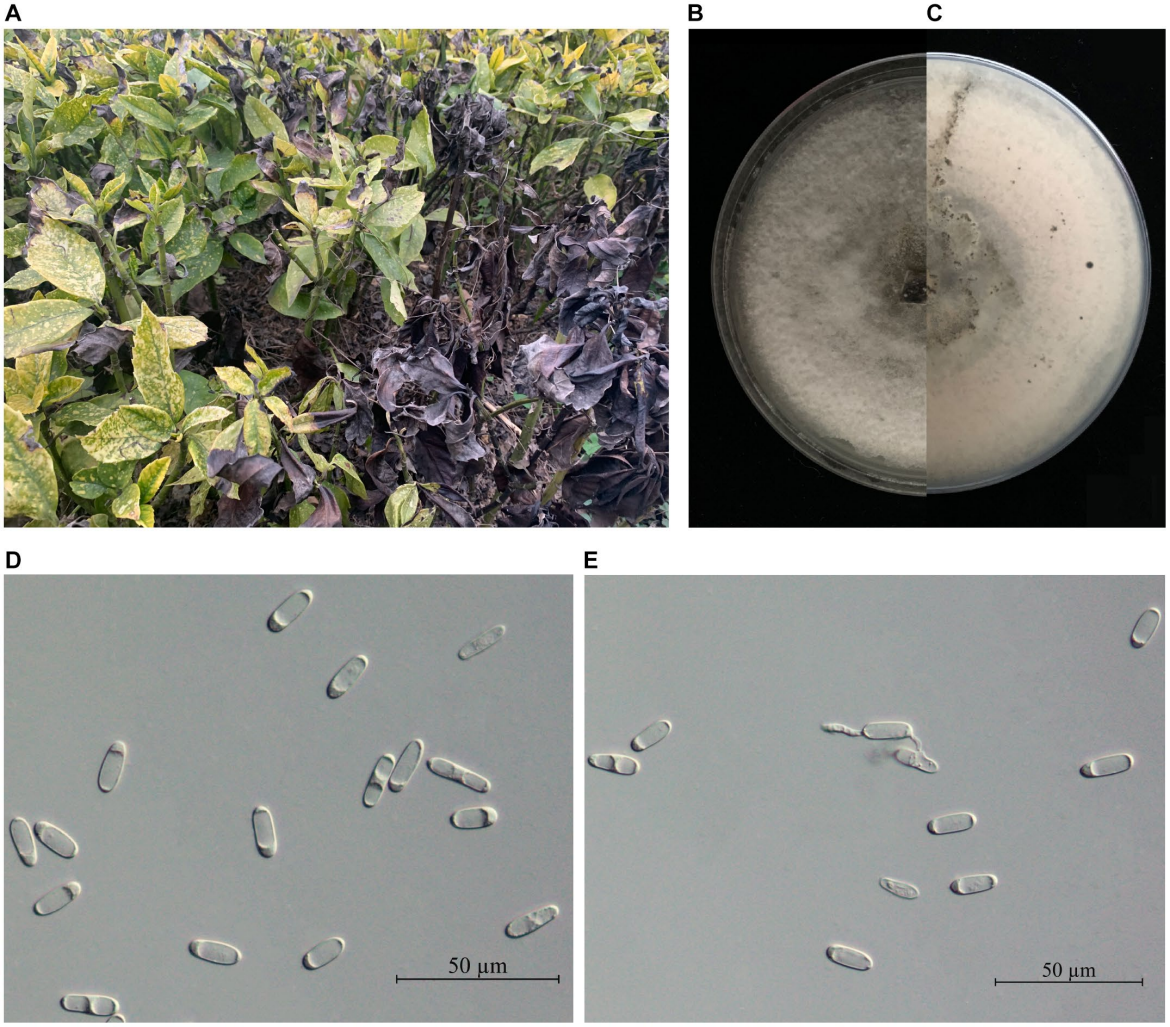
**(A)** Leaf blight disease of *Aucuba japonica* caused by *Colletotrichum* spp. **(B)** The front and **(C)** the reverse side of *C. aenigma* representative strain BY827 colonies were cultured on PDA for 7 days. **(D,E)** Conidia. Scale bars: D = 50 μm.

A total of 31 *Colletotrichum*-like isolates were obtained from 20 diseased *A. japonica* leaves (Figure 1A). The mycelial growth rate ranged from 4.7 to 5.0 mm/day. Initially, the colonies appeared white, but as time progressed, the reverse side of the colonies gradually turned pale gray from the center. The colonies were velutinous to woolly and had a regular circular shape (Figures 1B,C). Five representative strains were selected from the obtained isolates for microscopic observation. Conidia were cylindrical, with one end slightly acute or broadly rounded, and ranged in size from 13.67 to 21.09 μm × 4.24 to 7.30 μm (*n* = 60; Figures 1D,E). Based on these morphological characteristics, the isolates matched the description of the genus *Colletotrichum* spp. (Weir et al., 2012).

### 3.2. Molecular characterizations

**Figure 2:**
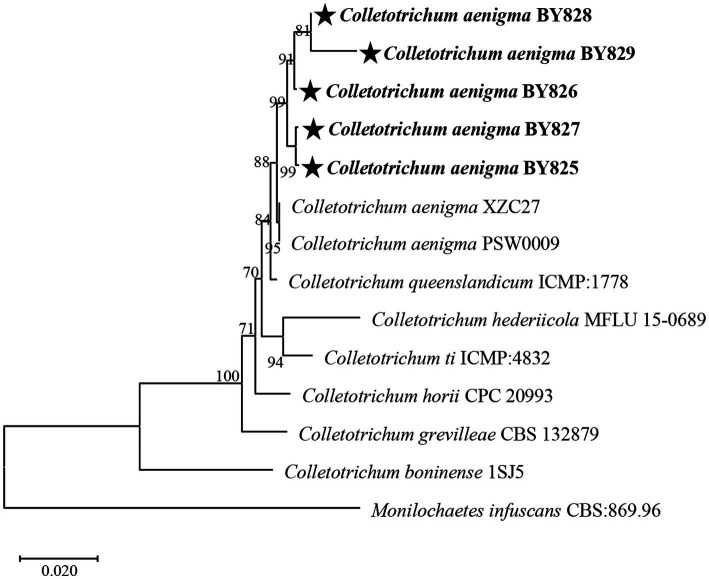
Neighbour-joining phylogenetic tree of concatenated sequences from the ITS region together with the chitin synthase (*CHS*), actin (*ACT*) genes of *Colletotrichum aenigma* BY825, *C. aenigma* BY826, *C. aenigma* BY827, *C. aenigma* BY828, and *C. aenigma* BY829 from this study and reference sequences of *Colletotrichum* spp. type materials. *Monilochaetes infuscans* CBS: 869.96 was used as the outgroup. Bootstrap values are provided next to the respective branches.

Nucleotide sequences were submitted for BLAST analysis using the NCBI-BLAST program. BLAST searches of the sequenced fragments resulted in the best match to *C. aenigma* sequences (Table 2). The sequences of the DNA regions of strains BY825, BY826, BY827, BY828, and BY829 were deposited in GenBank and are included in the Supplementary Material (Table 2). In addition, a phylogenetic analysis of the 5 strains was conducted, and the phylogenetic tree was constructed based on the ITS region, *CHS*, and *ACT* genes sequences (Figure 2). The results confirmed that the strains BY825, BY826, BY827, BY828, and BY829 belong to the *C. aenigma* group when compared with sequences of the type strains.

**Table 2:** Identification of isolates from *Aucuba japonica* leaves based on the comparison of ITS region, chitin synthase (*CHS*), and actin (*ACT*) sequences with the type strains in the database.

Isolate	Primer	GenBank accession number	Closely related type strain	Sequence similarity in NCBI (%)
BY825	ITS	OQ547224	*C. aenigma* (NR_120140.1)	99.63%
	*CHS*	OQ567717	*C. aenigma* (JX009774.1)	99.67%
	*ACT*	OQ567721	*C. aenigma* (JX009443.1)	99.64%
BY826	ITS	OQ547222	*C. aenigma* (NR_120140.1)	98.56%
	*CHS*	OQ567714	*C. aenigma* (JX009774.1)	99.26%
	*ACT*	OQ567718	*C. aenigma* (JX009443.1)	98.80%
BY827	ITS	ON521144	*C. aenigma* (NR_120140.1)	99.82%
	*CHS*	ON552999	*C. aenigma* (JX009774.1)	100.00%
	*ACT*	ON553000	*C. aenigma* (JX009443.1)	99.28%
BY828	ITS	OQ547223	*C. aenigma* (NR_120140.1)	99.82%
	*CHS*	OQ567715	*C. aenigma* (JX009774.1)	97.41%
	*ACT*	OQ567719	*C. aenigma* (JX009443.1)	99.59%
BY829	ITS	OQ547221	*C. aenigma* (NR_120140.1)	99.82%
	*CHS*	OQ567716	*C. aenigma* (JX009774.1)	99.24%
	*ACT*	OQ567720	*C. aenigma* (JX009443.1)	98.88%

### 3.3. Pathogenicity test

**Figure 3:**
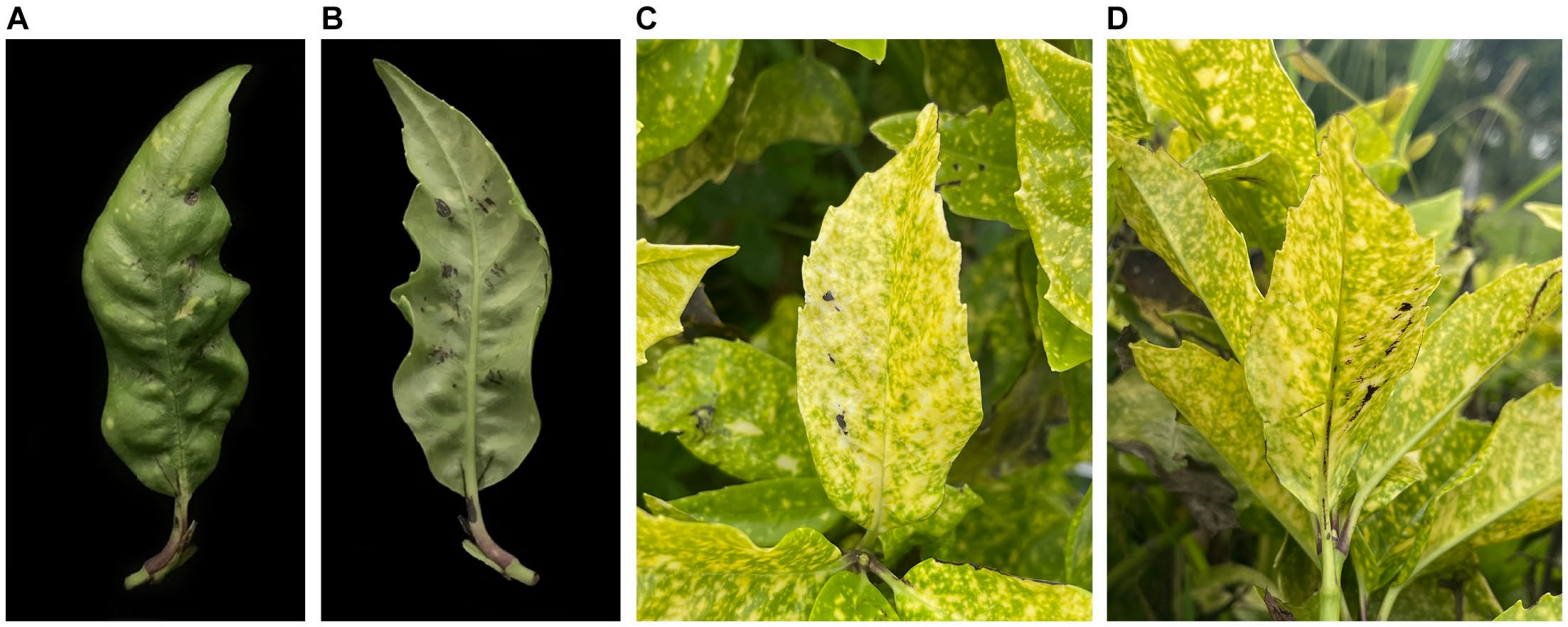
The leaf blight symptoms of *Colletotrichum aenigma* BY827 on *Aucuba japonica* detached leaves [**(A)** front side; **(B)** reverse side] and inoculated plants [**(C)** front side; **(D)** reverse side].

Seven days after inoculation, typical disease symptoms (early stage of blight: black spots) were visible on all inoculated plants (Figure 3). In contrast, leaves in the control group did not show any disease symptoms. The pathogenicity test was repeated and confirmed three times. Pure cultures were re-isolated from diseased leaves and confirmed to be *C. aenigma* BY827 based on the morphological and molecular methods mentioned above (ITS region, *CHS,* and *ACT* sequences).

### 3.4. Effect of temperature

**Figure 4:**
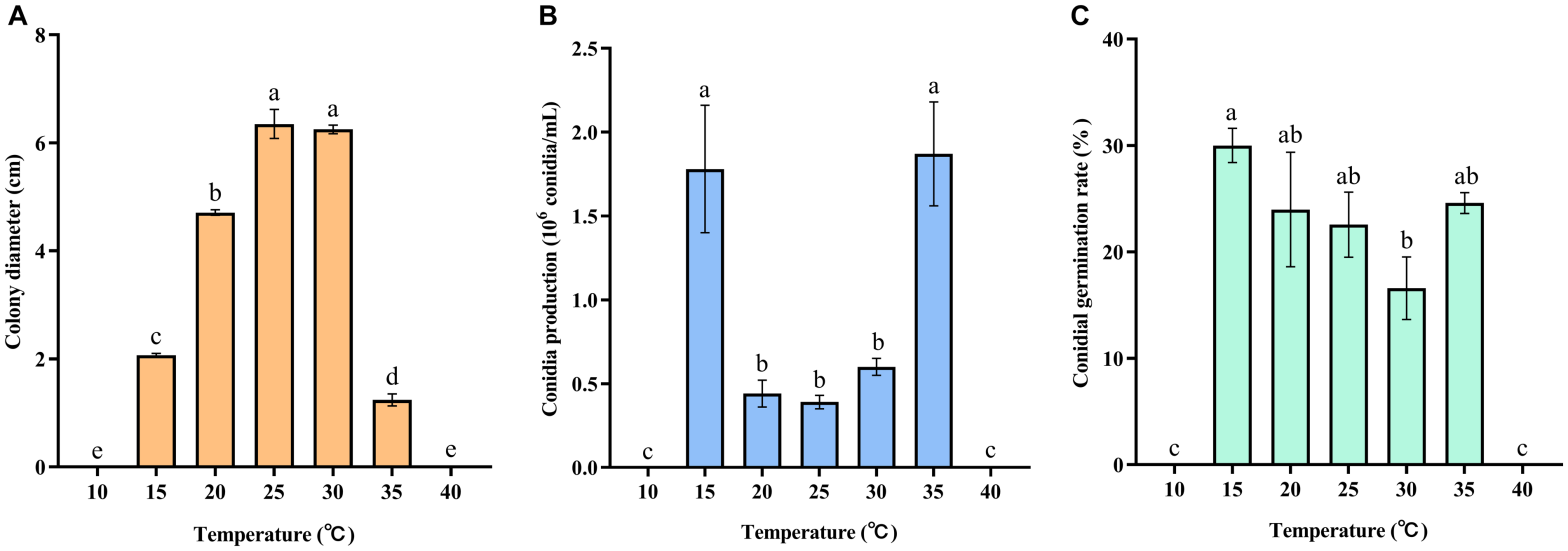
Effects of temperature on **(A)** mycelial growth, **(B)** conidia production, and **(C)** conidial germination rate of *Colletotrichum aenigma* BY827. Different lowercase letters indicate significant differences (*p* < 0.05). Data are mean ± SE.

The experimental results indicated that the pathogen was capable of growing within a temperature range of 15–35°C. The highest mycelial growth rate of *C. aenigma* BY827 was observed at 25°C and 30°C (Figure 4A). However, neither mycelium nor conidia could grow at 10°C and 40°C. The number of conidia produced by the pathogen was significantly higher at 15°C and 35°C, as shown in Figure 4B. Furthermore, the results showed that the conidial germination rate at 15°C was significantly higher at 30°C (Figure 4C).

### 3.5. Effect of photoperiod

**Figure 5:**
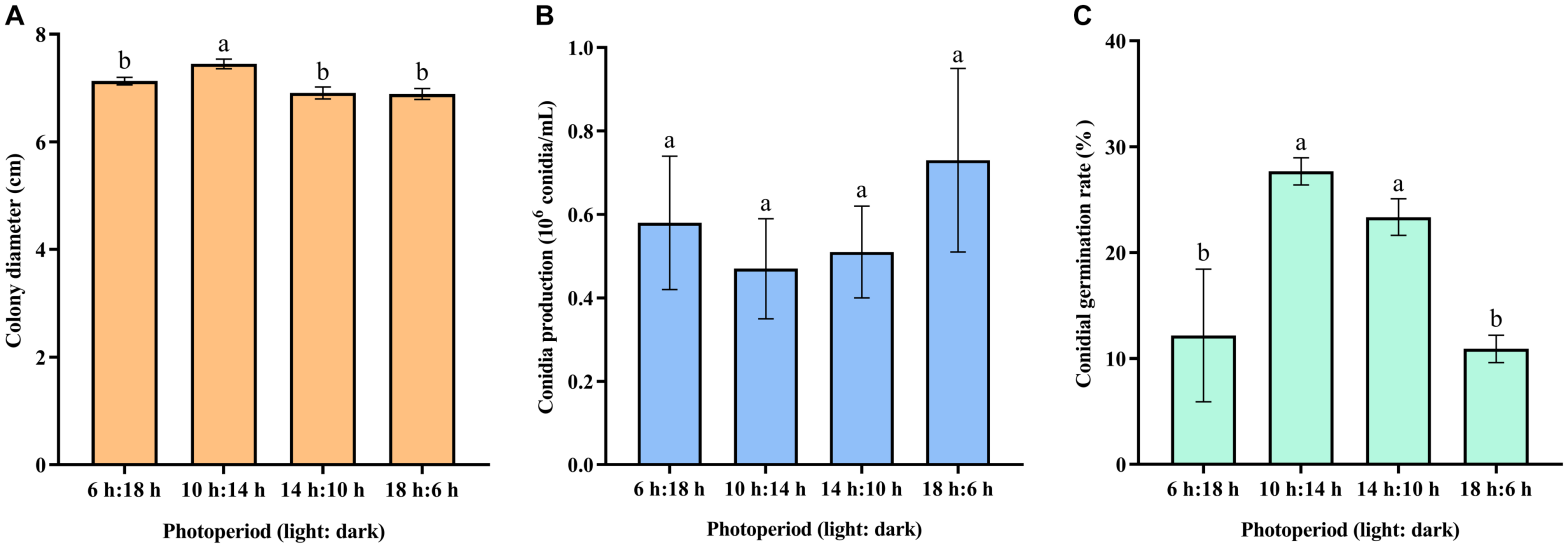
Effects of photoperiod on **(A)** mycelial growth, **(B)** conidia production, and **(C)** conidial germination rate of *Colletotrichum aenigma* BY827. Different lowercase letters indicate significant differences (*p* < 0.05). Data are mean ± SE.

The result indicated that 10-h/14-h alternating light increased the mycelial growth of *C. aenigma* BY827 (Figure 5A). There was no significant difference in conidia production under different photoperiods (Figure 5B). However, the alternation of 10 h light and 14 h dark, as well as 14 h light and 10 h dark, increased the conidial germination of *C. aenigma* BY827 (Figure 5C).

### 3.6. Effect of humidity

**Figure 6:**
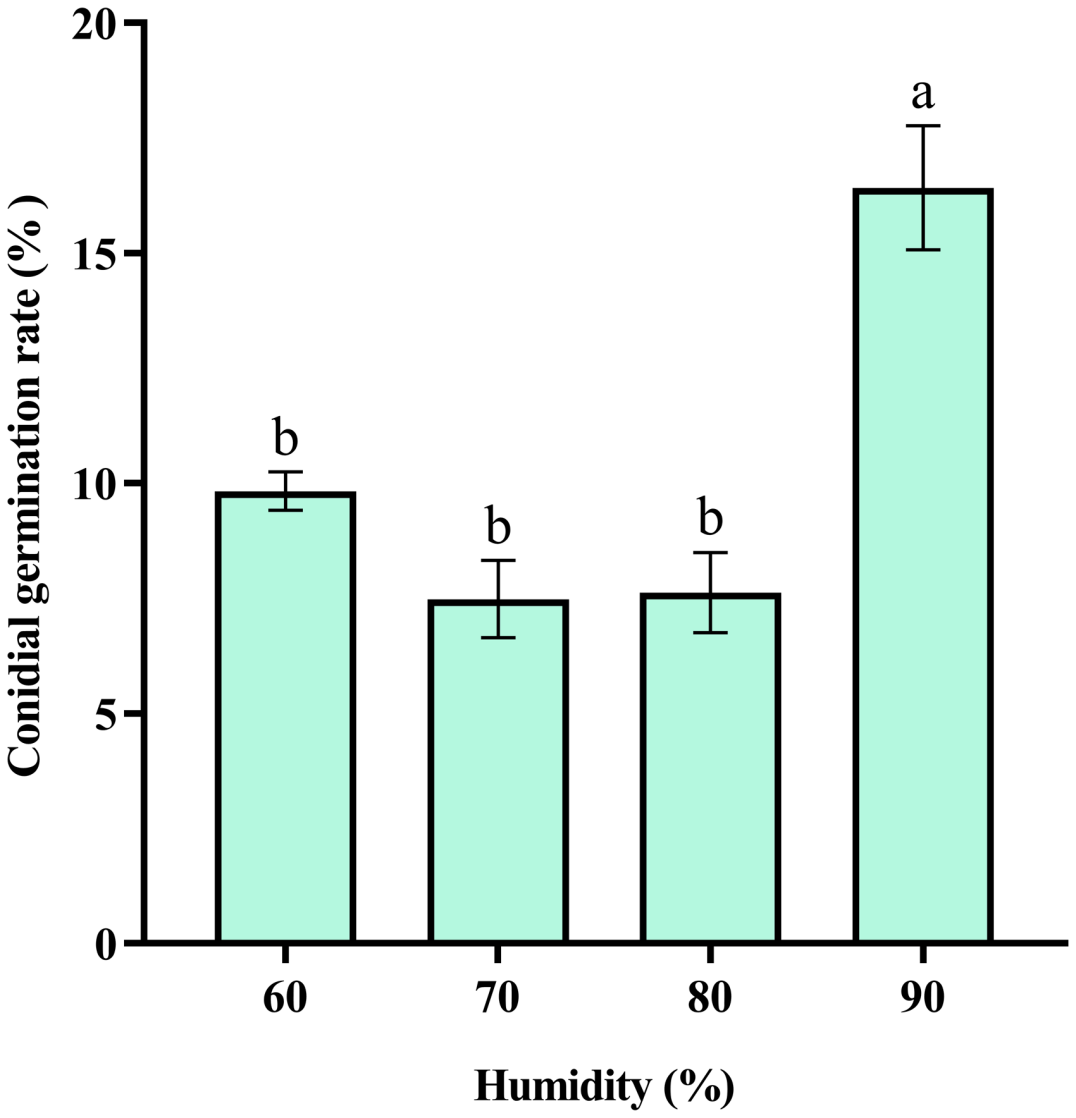
Effects of humidity on the conidial germination rate of *Colletotrichum aenigma* BY827. Different lowercase letters indicate significant differences (*p* < 0.05). Data are mean ± SE.

The results showed that at 90% humidity, the conidial germination rate of *C. aenigma* BY827 was significantly higher than that of other humidifies (Figure 6).

### 3.7. Fungicide assays

**Figure 7:**
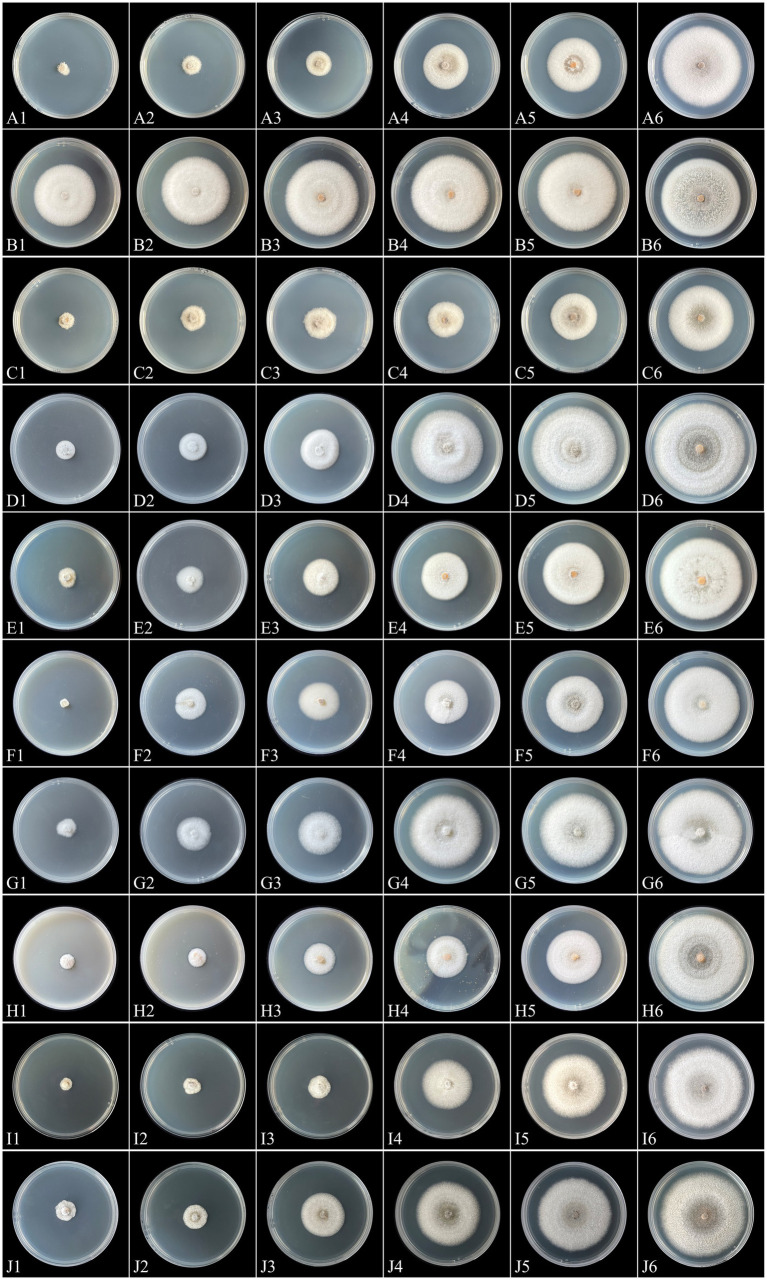
Mycelial growth of *Colletotrichum aenigma* BY827 on PDA plates incubated for 7 days in the absence (CK) or presence of different concentrations of tebuconazole (A1: 0.43 μg/mL, A2: 0.215 μg/mL, A3: 0.1075 μg/mL, A4: 0.05375 μg/mL, A5: 0.026875 μg/mL, A6: CK), mancozeb (B1: 6.88 μg/mL, B2: 3.44 μg/mL, B3: 1.72 μg/mL, B4: 0.86 μg/mL, B5: 0.43 μg/mL, B6: CK), difenoconazole (C1: 0.1 μg/mL, C2: 0.05 μg/mL, C3: 0.025 μg/mL, C4: 0.0125 μg/mL, C5: 0.00625 μg/mL, C6: CK), pentachloronitrobenzene (D1: 0.8 μg/mL, D2: 0.4 μg/mL, D3: 0.2 μg/mL, D4: 0.1 μg/mL, D5: 0.05 μg/mL, D6: CK), myclobutanil (E1: 1 μg/mL, E2: 0.5 μg/mL, E3: 0.25 μg/mL, E4: 0.125 μg/mL, E5: 0.0625 μg/mL, E6: CK), dimethachlone (F1: 12.8 μg/mL, F2: 3.2 μg/mL, F3: 0.8 μg/mL, F4: 0.2 μg/mL, F5: 0.05 μg/mL, F6: CK), hymexazol (G1: 2.8 μg/mL, G2: 1.4 μg/mL, G3: 0.7 μg/mL, G4: 0.35 μg/mL, G5: 0.175 μg/mL, G6: CK), thiram (H1: 8 μg/mL, H2: 4 μg/mL, H3: 2 μg/mL, H4: 1 μg/mL, H5: 0.5 μg/mL, H6: CK), carvacrol (I1: 1.6 μg/mL, I2: 0.8 μg/mL, I3: 0.4 μg/mL, I4: 0.2 μg/mL, I5: 0.1 μg/mL, I6: CK), and kasugamycin (J1: 1.28 μg/mL, J2: 0.64 μg/mL, J3: 0.32 μg/mL, J4: 0.16 μg/mL, J5: 0.08 μg/mL, J6: CK).

The sensitivities of the representative isolate *C. aenigma* BY827 to ten selected fungicides are presented in Table 3. Except for mancozeb, increasing concentrations of the fungicides inhibited the mycelial growth of *C. aenigma* BY827, as illustrated in Figure 7. Among the ten tested fungicides, the EC_50_ values of nine fungicides were less than 1 μg/mL. Difenoconazole showed the highest inhibition rate, with an EC_50_ value of 0.0148 μg/mL, followed by tebuconazole, pentachloronitrobenzene, myclobutanil, carvacrol, and kasugamycin, which also showed an effective inhibitory effect against *C. aenigma* BY827, with EC_50_ values of 0.0388 μg/mL, 0.1890 μg/mL, 0.1906 μg/mL, 0.2081 μg/mL, and 0.2996 μg/mL, respectively. In contrast, mancozeb did not show effective inhibitory effects against *C. aenigma* BY827, with EC_50_ values of 202.1461 μg/mL.

**Table 3:** Inhibitory effect of ten fungicides on *Colletotrichum aenigma* BY827.

Fungicide	Toxic regression equation	EC_50_ (μg/mL)	*r*	95% Confidence intervals	
Tebuconazole 43% SC	y = 1.3789x + 6.9458	0.0388	0.9907	0.0267	0.0506
Mancozeb 43% SC	y = 0.6637x + 3.4698	202.1461	0.8429	33.5009	142439.8350
Difenoconazole 10% WG	y = 1.0993x + 7.0099	0.0148	0.9935	0.0107	0.0191
Pentachloronitrobenzene 40% DP	y = 1.8838x + 6.3630	0.1890	0.9855	0.1620	0.2198
Myclobutanil 12.5% EC	y = 1.2414x + 5.8935	0.1906	0.9979	0.1492	0.2364
Dimethachlone 40% WP	y = 0.6391x + 5.2739	0.3728	0.9254	0.1007	0.9001
Hymexazol 70% WP	y = 1.7265x + 5.1445	0.8247	0.9780	0.7020	0.9793
Thiram 50% WP	y = 1.2316x + 5.1310	0.7827	0.9883	0.5301	1.0304
Carvacrol 5% AS	y = 1.8539x + 6.2638	0.2081	0.9717	0.1667	0.2488
Kasugamycin 2% AS	y = 1.7119x + 5.8960	0.2996	0.9855	0.2535	0.3527

### 3.8. Field experiments

The results of the field experiment were presented in Table 4. Difenoconazole exhibited the highest control efficiency with a decreasing rate of disease incidence of 44.60% and decreasing rate of disease index of 47.75%, respectively, which were significantly higher than other treatments. Tebuconazole and pentachloronitrobenzene showed no differences in disease incidence and disease index. Moreover, mancozeb exhibited the lowest control efficiency with decreasing rate of disease incidence of 3.53% and decreasing rate of disease index of 3.77%, respectively, which were significantly lower than other treatments.

**Table 4:** Control effect of four fungicides on the leaf blight of *Aucuba japonica* in field experiments.

Treatments	Before treatment		After treatment		Control efficiency (decreasing rate; %)	
	Disease incidence (%)	Disease index	Disease incidence (%)	Disease index	Disease incidence (%)	Disease index (%)
Control	46.20 ± 2.31 a	34.29 ± 1.90 a	46.12 ± 2.63 a	33.07 ± 1.64 a	–	–
Tebuconazole 43% SC	44.19 ± 1.38 a	34.25 ± 1.47 a	32.18 ± 2.42 b	24.03 ± 1.35 bc	27.36 ± 3.17 b	26.97 ± 5.59 b
Mancozeb 43% SC	47.46 ± 0.31 a	32.45 ± 1.41 a	45.79 ± 0.49 a	30.13 ± 1.24 a	3.53 ± 0.41 c	3.77 ± 0.63 c
Difenoconazole 10% WG	48.79 ± 4.23 a	38.73 ± 4.25 a	27.19 ± 3.80 b	19.29 ± 1.61 c	44.60 ± 5.16 a	47.75 ± 4.75 a
Pentachloronitrobenzene 40% DP	46.26 ± 4.19 a	32.79 ± 1.27 a	36.37 ± 3.30 b	24.64 ± 1.75 b	21.14 ± 4.68 b	22.05 ± 5.19 b

Data are presented as the means ± SE. Different letters in the same column indicate statistical significance (*p* < 0.05).

## 4. Discussion

Globally, *Colletotrichum* spp. are well-known for their high pathogenicity in causing anthracnose, a serious leaf blight disease that results in significant yield loss or quality reduction in various plants, including agricultural crops, fruit trees, and ornamental shrubs, across different ecosystems (Sharma et al., 2014). Previously, *C. boninense* was confirmed as the causative agent of anthracnose in *A. japonica* (Liu et al., 2022). In our study conducted in Guiyang, Guizhou, China, we isolated and identified *C. aenigma* as the causal agent of leaf blight disease in *A. japonica*. To our knowledge, this is the first report of *C. aenigma* causing leaf blight on *A. japonica* worldwide. This identification may help decision-makers to establish targeted control measures against the disease.

Understanding the biological characteristics of phytopathogens is crucial for the efficient control of related plant diseases. The results of the biological characteristics study showed that the optimum temperature for *C. aenigma* BY827 mycelial growth was 25–30°C. This was similar to other pathogenic *Colletotrichum* spp., such as *C. truncatum* and *C. camelliae*, optimum mycelial growth temperatures ranged from 25.0–29.3°C (Lu et al., 2018; Júnior et al., 2021). In addition, *C. aenigma* BY827 could not grow above 40°C condition, which is consistent with *C. asianum* T0408, another pathogenic strain caused mango anthracnose (Sun et al., 2016). The mycelial growth and conidial germination rate of *C. aenigma* BY827 were higher under the photoperiod of 10 h light and 14 h dark, which aligns with the local photoperiod conditions of Guiyang City, Guizhou Province, China (The climate data were compiled from the China Meteorological Data Network).^2^ Moreover, the conidial germination rate of *C. aenigma* BY827 increased when the relative humidity reached 90%, which is consistent with *C. gloeosporioides* I-2, also the causal agent of anthracnose disease on mango (Estrada et al., 2000).

Currently, chemical control is the most common and widely applied strategy for controlling plant diseases caused by *Colletotrichum* spp. (Yin et al., 2018). However, the long-term and extensive use of chemical fungicides resulted in various threats, including concerns about food safety, environmental pollution, and adverse effects on human health (Chen et al., 2021). Moreover, the development of resistance by pathogenic fungi is one of the main reasons for the resurgence of diseases, and residual issues of pesticides (Fair and Tor, 2014; Duan et al., 2020; Black et al., 2022; Tang et al., 2022). Therefore, it is necessary to screen effective pesticides for managing newly emerging diseases, such as leaf blight of *A. japonica* caused by *C. aenigma*. In our study, we conducted *in vitro* screening of ten different pesticides (eight chemical fungicides, and two biopesticides widely used to control pathogens like *Colletotrichum* spp.), and performed field trials of four fungicides (selected based on their effectiveness in the *in vitro* screening) against *C. aenigma* (Morsy and Elshahawy, 2016; Zhang et al., 2017; Fan et al., 2022). The results consistently showed that three pesticides, tebuconazole, difenoconazole, and pentachloronitrobenzene, significantly suppressed *C. aenigma* BY827 both *in vitro* and *in vivo*.

Furthermore, difenoconazole exhibited the highest inhibition rate, with an EC_50_ value of 0.0148 μg/ml, followed by tebuconazole and pentachloronitrobenzene. Similar results were observed for difenoconazole when used against its target pathogen *Aspergillus fumigatus* (Schoustra et al., 2019). Moreover, difenoconazole exhibited the highest control efficiency (47.75%), indicating potent antifungal activity against *Colletotrichum* spp. (Zhang et al., 2020; Peng et al., 2022). In contrast, mancozeb showed no potent inhibitory activity against *C. aenigma* BY827, with EC_50_ values of 202.1461 μg/mL. Mancozeb is a broad-spectrum fungicide that normally exhibits good inhibitory effects against fungi pathogens. For example, it significantly suppressed the tuber blight pathogen *Pyricularia oryzae* under both laboratory and field conditions (Kongcharoen et al., 2020). Until now, very few pathogens have been reported to be resistant to mancozeb (Tang et al., 2022). Torres-Calzada et al. (2015) reported that there was no mancozeb resistance in *Colletotrichum truncatum* isolates in Mexico. However, our study found that *C. aenigma* BY827 may have developed resistance to mancozeb, which was confirmed in field experiments. Our research indicated that mancozeb exhibited the lowest control efficiency (the decreasing rate of disease incidence and decreasing rate of disease index were 3.53 and 3.77%, respectively) and was significantly less effective than other fungicides. Furthermore, the intensive or overuse of mancozeb in such circumstances could increase the risks of environmental issues, especially in sensitive ecosystems like the karst landscapes.

In summary, our study provided new insights into the occurrence of *A. japonica* leaf blight disease in Guiyang, Guizhou, China, and identified *C. aenigma* as the new causal agent. Moreover, we evaluated the influences of crucial environmental factors for ornamental plant production, including temperature, photoperiod, as well as humidity, on the biological characteristics of representative strain *C. aenigma* BY827. Interestingly, we found that the biological characteristics of *C. aenigma* BY827 showed good adaptions to the local environmental conditions of Guiyang City. These could be taken into consideration for its future management. Furthermore, the results of *in vitro* fungicide screening assays and field experiments provided effective fungicide candidates, including difenoconazole, pentachloronitrobenzene, and tebuconazole for the control of *A. japonica* leaf blight caused by *C. aenigma*. Further studies could include the assessing of practical control efficiencies of these fungicides against *C. aenigma* on *A. japonica* plants in large-scale and in different application scenarios. In addition, monitoring mancozeb resistance among *C. aenigma* isolates, as well as possible cross-resistance between other pathogen populations, would provide valuable information on the evolution of fungicide resistance in unique ecosystems like karst landscapes in Guizhou, China. As a whole, the main findings from our study could contribute to developing future sustainable and effective management strategies to control *A. japonica* leaf blight disease caused by *C. aenigma*, especially in Guiyang, Guizhou, China.

## Data availability statement

The datasets presented in this study can be found in online repositories. The names of the repository/repositories and accession number(s) can be found in the article/supplementary material.

## Author contributions

XC and XH designed the experiments. RF, YjL, YB, JH, BY, XT, YxL, YC, ZY, MY, JS, and QP performed the experiments. RF, YjL, YB, MIG, and XC drafted the manuscript. RF, YB, YjL, and XH analyzed data. RF, XH, ZL, MIG, and XC conducted visualization and proofreading of the manuscript.

## Funding

This study was supported by National Key Research and Development Program of China (2021YFE0107700), Science and Technology Base and Talent Project of Guangxi Province (Guike AA21196003), Guizhou Provincial Science and Technology Program (2019-1410; HZJD[2022]001; 2021-229), Outstanding Young Scientist Program of Guizhou Province (KY2021-026), Guizhou University Cultivation Project (2019-04; 2022-085; SYSKF2023-093), and Program for Introducing Talents to Chinese Universities (111 Program; D20023).

## Conflict of interest

The authors declare that the research was conducted in the absence of any commercial or financial relationships that could be construed as a potential conflict of interest.

## Publisher’s note

All claims expressed in this article are solely those of the authors and do not necessarily represent those of their affiliated organizations, or those of the publisher, the editors and the reviewers. Any product that may be evaluated in this article, or claim that may be made by its manufacturer, is not guaranteed or endorsed by the publisher.

## Footnotes

^1^ http://www.ncbi.nlm.nih.gov/

^2^ http://data.cma.cn/
